# Faecal D/L Lactate Ratio Is a Metabolic Signature of Microbiota Imbalance in Patients with Short Bowel Syndrome

**DOI:** 10.1371/journal.pone.0054335

**Published:** 2013-01-23

**Authors:** Camille Mayeur, Jean-Jacques Gratadoux, Chantal Bridonneau, Fatima Chegdani, Béatrice Larroque, Nathalie Kapel, Olivier Corcos, Muriel Thomas, Francisca Joly

**Affiliations:** 1 Service de Gastroentérologie et Assistance Nutritive, Pôle des Maladies de l'Appareil Digestif, Hôpital Beaujon, Clichy, France; 2 INRA, UMR1319 MICALIS, Commensal and Probiotics-Host interactions Laboratory, Jouy-en-Josas, France; 3 AgroParisTech, UMR1319 MICALIS, Commensal and Probiotics-Host interactions Laboratory, Jouy-en-Josas, France; 4 Epidemiological and Clinical Research Unit, Hôpital Beaujon, Clichy, France; Instutite of Agrochemistry and Food Technology, Spain

## Abstract

Our objective was to understand the functional link between the composition of faecal microbiota and the clinical characteristics of adults with short bowel syndrome (SBS).

Sixteen patients suffering from type II SBS were included in the study. They displayed a total oral intake of 2661±1005 Kcal/day with superior sugar absorption (83±12%) than protein (42±13%) or fat (39±26%). These patients displayed a marked dysbiosis in faecal microbiota, with a predominance of *Lactobacillus/Leuconostoc* group, while *Clostridium* and *Bacteroides* were under-represented. Each patient exhibited a diverse lactic acid bacteria composition (*L. delbrueckii* subsp. *bulgaricus*, *L. crispatus*, *L. gasseri*, *L. johnsonii*, *L. reuteri*, *L. mucosae*), displaying specific D and L-lactate production profiles *in vitro*. Of 16 patients, 9/16 (56%) accumulated lactates in their faecal samples, from 2 to 110 mM of D-lactate and from 2 to 80 mM of L-lactate. The presence of lactates in faeces (56% patients) was used to define the Lactate-accumulator group (LA), while absence of faecal lactates (44% patients) defines the Non lactate-accumulator group (NLA). The LA group had a lower plasma HCO3^−^ concentration (17.1±2.8 mM) than the NLA group (22.8±4.6 mM), indicating that LA and NLA groups are clinically relevant sub–types. Two patients, belonging to the LA group and who particularly accumulated faecal D-lactate, were at risk of D-encephalopathic reactions. Furthermore, all patients of the NLA group and those accumulating preferentially L isoform in the LA group had never developed D-acidosis. The D/L faecal lactate ratio seems to be the most relevant index for a higher D- encephalopathy risk, rather than D- and L-lactate faecal concentrations *per se*. Testing criteria that take into account HCO3^−^ value, total faecal lactate and the faecal D/L lactate ratio may become useful tools for identifying SBS patients at risk for D-encephalopathy.

## Introduction

Micro-organisms colonizing the digestive tract (microbiota) play a key role in nutrition and health. The diversity and abundance of this microbial population makes it difficult to define a “healthy intestinal flora”. In general, adult human microbiota is mainly composed of a phylogenic core containing *Firmicutes*, *Bacteroidetes*, and *Actinobacteria* [1], [2]. It is well known that the composition and mostly, molecular functions of microbiota are altered by age, nutrition, environment and pathologic status [3], [4], [5], [6]. Dysbiosis is characterised by a divergent ratio between the major groups and/or by the emergence of specific microorganisms. The protective or harmful role of bowel microbiota depends on the pathology, and thus microbiota specifically influences the aetiopathogenesis of each human disease.

In patients with type II short bowel syndrome (SBS), the aetiopathogenesis has been associated with an extreme dysbiosis [7], [8], [9], [10]. These patients have had a massive small bowel resection, leaving less than 150 cm joining with a partial or an entire colon [11]. These patients have such a reduced intestinal surface that they suffer from major water and nutrient mal-absorption, requiring oral and parenteral nutrition [12], [13], [14], [15]. Microbiota of SBS patients is markedly disturbed with a high prevalence of *Lactobacillus/Leuconostoc* group along with a drop in numbers and a poor diversity of *Clostridium leptum*, *Clostridium coccoides* and *Bacteroides* [7], [9]. Bacterial groups abundant in healthy humans nearly disappear in SBS, while *Lactobacillus/Leuconostoc* group, sub-dominant in healthy controls, become over-represented in SBS [9]. In addition to these unusual microbial features, we previously detected a SBS-associated species, *Lactobacillus mucosae* which has never been found in healthy controls [9]. Intestinal resection probably favours the *Lactobacillus/Leuconostoc* group and the emergence of *L. mucosae* because they are aerobic-tolerant micro-organisms and moreover these bacteria resist the acidic environment and nutrient load, using mal-absorbed nutrients as an energy source. The Roux-en-Y gastric bypass (RYGB), a current surgical treatment for morbid obesity, is also associated with changes in microbial composition and activities [16], [17]. Short bowel syndrome and gastric bypass illustrate how gut remodelling due to surgery impacts the microbiota that in turn becomes central to understanding the clinical outcome.

In SBS patients, it has been shown that the presence of colon determines decreased dependence on parenteral nutrition. The number of calories provided by microbiota in healthy humans was up to 150 Kcal [18]; since the colonic digestion *via* microbiota activity may provide an energy supply of up to approximately 1000 kcal per day in SBS [19], [20], [21]. The remaining colon adapts following resection by increasing cell numbers and crypt depth. It plays a key role to salvage energy *via* the fermentative activities of endogenous microbiota [22]. Some SBS patients develop severe, symptomatic D-lactic acidosis, with metabolic acidosis and neurological disorders. The D-lactic acidosis is linked to both the metabolic activity of microbiota and the individual ability to metabolize D-lactic acid [23], [24], [25]. The occurrence of D-lactic acidosis remains sporadic and non-predictable. D-lactic acidosis may be more common than is thought and should be looked for in cases of metabolic acidosis in which the cause of acidosis is not apparent. The clinical presentation is often characterised by episodes of unusual neurological manifestations and severe metabolic acidosis. Plasmatic bicarbonate assays are done routinely to monitor SBS patients and evaluate ionic balance. But, the relationship between neurological disorders, microbiota composition, fermentative activity, metabolism and systemic acidosis has not been yet completely understood [7], [26]. Moreover, a survey of SBS patients is difficult since measurement of serum D-lactate concentration is not routinely available in most hospital laboratories or the results come back after the D-lactic acidosis crisis [27], [28]. Our objective was to understand the functional link between the composition/metabolic activity ratio of the faecal microbiota and the SBS clinical characteristics.

## Methods

### SBS Patients

Ethics statement: The Human Investigations Committee of Hospital Saint-Louis in Paris approved the study (n: 031048) in January 2004. All patients gave their written informed consent to participate in this study.

This prospective study was performed between January 2006 and January 2011. Patients were enrolled and monitored in our nutrition support unit, an approved centre for intestinal failure and Home Parenteral Nutrition, located at Lariboisiere and Beaujon hospitals (Paris and Clichy respectively). Clinical and demographic information were collected from the case records. Data included: sex, age, and medical history, SBS aetiology, characteristics of past surgical procedures, and characteristics of intestinal tract. All patients received gastric anti-secretory drugs (IPP) but none had pancreatic enzyme treatment and cholestyramine.

### Inclusion criteria

- SBS type II with a post duodenal remnant jejunum <150 cm, no remnant ileum and re-established jejuno-colic continuity for more than 2 years.
- a non restricted oral diet.

Exclusion criteria:

- upper gastrointestinal tract surgery (oesophagus, stomach, duodenum and/or pancreas).
- a remnant ileum.
- radiation enteritis and active Crohn's disease,
- organ failure other than gastrointestinal,
- evolutive neoplasia.
- intestinal fistulae.
- treatment by recombinant human growth hormone or glucagon like peptide-2 in the last 12 months.
- BMI>30.
- age >80 or <18 years.
- sepsis and/or antibiotics or aspirin in the 4 weeks before the study.
- treatments with steroids or immunosuppressive therapy in the 4 months preceding the study.
- pregnancy
- a D-lactic encephalopathy episode in the 3 months preceding the study.

The remaining colon in continuity, expressed in terms of percent of the usual length according to the method of Cummings *et al.*, was estimated on the basis of per-operative records [29].

#### General procedures

Following patient screening, eligible patients were invited to participate in the study during their regular out-patient check up. When patients had given their informed consent, biological analyses were performed.

### Dietary

The patients were asked to maintain their usual intake. Under the supervision of trained dieticians, patients noted the type, amount or weight of foods they ingested at each meal, including snacks and nibbling for 3 days during the week preceding sample collection. The daily intake of total calories, proteins, carbohydrates (total, complex and simple), fat, fibre and alcohol were calculated with Bilnut® software (Bourgerette P, Rolshansen M. BILNUT 4.0. SCDA Nutrisoft. Le Hallier 37390 Cerellers, France). The mean intake for each macronutrient and fibre were expressed as the percentage of total g/day oral intake.

### Absorption study

Over a 6-day period, the first 3-day baseline period confirmed that patients were continuing their spontaneous intake of energy, carbohydrates, fat, protein and fibres. Absorption was measured during the following 3 days. Stools were collected daily and frozen at −20°C. Protein, lipid, and total energy were determined by nitrogen elemental analysis (N analyser Flash EA1112, Thermo Scientific, Waltham, MA) [30], and the method of Van de Kamer and bomb calorimetry (PARR 1351 bomb calorimeter, Parr instrument company, Moline, IL) [31], respectively. Quantification of carbohydrate-derived energy was calculated by subtracting the energy associated with the protein and lipid components from the total energy. The calorie-conversion factors used were 4.2, 9.35 and 5.65 kcal/g for carbohydrates, lipids and proteins respectively [32]. The coefficient of net intestinal absorption represented the proportion of ingested energy not recovered in stool output. REE kcal/day (resting energy expenditure): basal metabolic rate assessed with Harris and Benedict equations ×1.5 required to obtain energy balance equilibrium in patients with short bowel syndrome.

### Biological analysis

Routine biological data included serum albumin, transthyretin, electrolytes and C Reactive Protein (CRP). The anion gap was calculated from measured electrolytes as the sum of serum chloride and bicarbonate concentrations subtracted from the sodium and potassium concentrations [(Na^+^+K^+^)-(Cl^−^+HCO3^−^)]. Faecal samples (200 mg fractions) were stored in sterile tube at −80°C until further processing.

### D-encephalopathy description

We considered a patient having a past history of D-lactic encephalopathy when characteristic clinical presentation of recurrent episodes of encephalopathy was associated or not with metabolic acidosis. The symptoms and signs included altered mental status, slurred speech, ataxia, gait disturbance, confusion, weakness, and/or impaired motor coordination. Hostile, aggressive, and abusive behaviour as well as inability to concentrate are also characteristic neurologic features. Nystagmus is sometimes a sign of the disease. The correction of neurological symptoms by fasting is an additional diagnostic element.

### Bacterial strains and culture conditions

*Lactobacillus delbrueckii* subsp. *bulgaricus* ATCC 11842 (ATCC collection, USA), *Lactobacillus crispatus* DSM 20584, *Lactobacillus gasseri* DSM 20243T, *Lactobacillus reuteri* DSM 20016, *Lactobacillus mucosae* DSM 13345 (DSMZ collection, Germany) and *Lactobacillus johnsonii* VEL 12201 (MICALIS collection, INRA, France) were used. Bacteria were grown in MRS - 0.5 g/l Cystein(Cys) medium at 37°C in anaerobic conditions (Anaerocult A, Merk, Darmstadt, Germany).

### D and L-Lactate analyses on in vitro Lactobacillus cultures and faeces

D and L-lactates were measured from different *Lactobacillus* species *in vitro* cultures and directly from stools. The medium used for the pre-cultures and cultures of *Lactobacillus* was MRS supplemented with 0.5 g/l Cys. A preculture of each species was platted on solid medium and then, individual colony of each strain was grown in MRS-Cys at 37°C in anaerobic conditions (Anaerocult A) during 24 hours. D and L-lactates were measured on 24 h *in vitro* culture from each strain using 1 ml of culture supernatant firstly precipitated with TCA 6 N (10%). For faecal dosage, 100 to 250 mg frozen stools were 2 fold diluted in 0.1 M triethanolamine buffer (pH 9.15) and precipitated with TCA 6 N (10%). D and L-Lactates were measured in TCA-supernatants using Biosentec D/L lactic acid enzymatic kit according to manufacturer instructions (Biosentec, Toulouse, France) and as we described previously [33].

### Identification of bacterial groups that were quantified by real-time qPCR

The dominant groups (*C. leptum*, *C. coccoides*, *Bacteroides/Prevotella*, *Bifidobacterium*, *E. coli and Lactobacillus/Leuconostoc*) were quantified by real-time qPCR using primers and probes (Table 1), as previously described [34], [35], [36], [37]. Amplification efficiencies in real-time qPCR of all taqman and sybergreen systems were reported in Table 1.

**Table 1:** All 16S targeted primers and probes used in this study.

	Target	Primers and probe	Sequences (5′-3′)	Associated reference strain	Product Size (bp)	Amplification efficiency %	Reference
**TAQMAN SYSTEM**	All bacteria	F-Bact 1369	CGGTGAATACGTTCCCGG	*E. coli*			
		R-Prok1492	TACGGCTACCTTGTTACGACTT	UEPSD S123		90–99	37
		**P-TM1389F**	**6 FAM –CTTGTACACACCGCCCGTC**				
	*Clostridium leptum* group	F-Clept09	CCTTCCGTGCCGSAGTTA	*C. leptum*			
		R-Clept 08	GAATTAAACCACATACTCCACTGCTT	DSM 753		82–97	34
		**P-Clep 01**	**6 FAM-CACAATAAGTAATCCACC**				
	*Clostridium coccoides* group	F-Ccoc07	GACGCCGCGTGAAGGA	*C. coccoides*			
		R-Ccoc14	AGCCCCAGCCTTTCACATC	ATCC 29236		85–99	34
		**P- Erec482**	**VIC-CGGTACCTGACTAAGAAG**				
	*Bacteroides/Prevotella* group	F-Bacter11	CCTWCGATGGATAGTGGTT	*B. fragilis*			
		R-Bacter 08	CACGCTACTTGGCTGGTTCAG	DSM 2151T		85–95	34
		**P- Bac303**	**VIC-AAGGTCCCCCACATTG**				
	*Bifidobacterium* group	F-Bifid 09c	CGGGTGAGTAATGCGTGACC	*B.adolescentis*			
		R-Bifid 06	TGATAGGACGCGACCCCA	DSM 20083		85–90	34
		**P- Bifid**	**6 FAM-CTCCTGGAAACGGGTG**				
**SYBERGREEN SYSTEM**	*Lactobacillus/Leuconostoc/*	F-lacto05	AGCAGTAGGGAATCTTCCA	*L. acidophilus*			
	*Pediococcus* group	R-Lacto04	CGCCACTGGTGTTCYTCCATATA	VEL 12085	547	80–85	34
	*Escherichia coli*	F- Ecoli F	CATGCCGCGTGTATGAAGAA	*E. coli*			
		R- Ecoli R	CGGGTAACGTCAATGAGCAAA	UEPSD S123	95	85–90	36
	*Streptococcus*	F- Sterm03	TTATTTGAAAGGGGCAATTGCT	*S. salivarius*			
		R- Sterm08	GTGAACTTTCCACTCTCACAC	ATCC 9222	281	85–95	34
	*Lactobacillus mucosae*	F- L.muc01	CGTTGGCCCAACTGATTGAA	*L. mucosae*			
		R- L.muc04	TATGCGGTATTAGCATCTGTTTCC	DSM 13345	137	88–95	9
	*Lactobacillus delbrueckii*	F- L.delb03	CAAGTCGAGCGAGCTGAATTC	*L.delbrueckii*			
	subsp. *bulgaricus*	R- L. delb02	GACAGCTTACGCCGCCTTTC	subsp. *bulgaricus*	182	89–95	This study
				ATCC 11842			
	*Lactobacillus crispatus*	F- L. crisp01	TTCGGTAATGACGTTAGGAAAGC	*L. crispatus*			
		R- L. crisp02	CCGGTATTAGCACCTGTTTCCA	DSM 20584	114	93–95	This study
	*Lactobacillus*	F-L.gasse03	AGTCGAGCGAGCTAGCCTAGATG	*L. gasseri*			
	*gasseri/johnsonii*	R-L.gasse02	AGCATCTGTTTCCAGGTGTTATCC	DSM 20243T	137	85–90	This study
	*Lactobacillus reuteri*	F- L.reuter07	CGTACGCACTGGCCCAA	*L. reuteri*			
		R- L.reuter06	TATGCGGTATTAGCATCTGTTTCC	DSM 20016	143	90–95	This study

All new *Lactobacillus* primers were designed and used in sybergreen system as described in materials and methods.

The *Lactobacillus* species, quantified by real-time qPCR was chosen following our previous results obtained by sequencing a “Lactobacillus/Leuconostoc” amplicon. This amplicon was obtained by amplifying faecal DNA with primers of the V6–V8 region designed on the 16S ribosomal DNA from *Lactobacillus/Leuconostoc group*. Primers (Lac 352-f and Lac 679-GC-r) and PCR conditions used were described previously [9], [38], [39] The PCR amplicon was cloned into TOPO4 (invitrogen, Cergy-Pontoise, France) and 40 clones were sequenced. Sequences were compared to known sequences in the Ribosomal Database Project using Blast2seq and Seqmatch programs. The amplicon sequences detected with high blast hits (95 to 99% identity) were : *L. delbrueckii* subsp. *bulgaricus*, *L. crispatus, L.gasseri, L. mucosae, L. reuteri.*

### Lactobacillus-specific primer design

Primers targeting different *Lactobacillus* species (*L. mucosae*, *L. delbrueckii* subsp. *bulgaricus, L. crispatus, L. gasseri/johnsonii, L. reuteri*) were based on specific region of the 16S ribosomal DNA sequences aligned using the Blastn program of NCBI database and design using Primer-express Version 2.0 (Applied-biosystems) (Table 1). Primer specificities were challenged *in silico* against sequences in Ribosomal Database project II using the probe match program. Primer specificities were then firstly assessed by real-time qPCR amplification against DNA from dominant faecal species or closely related species and secondly using PCR amplification followed by visualisation on agarose gel (Table S1). Amplification and detection were carried out in 96-well plates with Syber-Green PCR 2× master mix (Applied-Biosystems).

### Experimental conditions of real-time qPCR

Total bacterial DNA was extracted from 0.15 g to 0.2 g of faecal samples using a bead-beating method G'NOME DNA extraction kit (Mp Biomedicals, Illkirch) as described by Furet *et al* [34]. Faecal DNAs were dissolved in 150 µl Tris-EDTA buffer and stored at −20°C.

The real-time qPCR was performed using the ABI7000 sequence detection system apparatus and 7000 system software version 1.2.3 (Applied-Biosystems). Amplification and detection were carried out in 96-well plates with Taqman universal 2×PCR master mix or with Syber-Green PCR 2× master mix (Applied-Biosystems). Each reaction was run in duplicate in 25 µl with 0.2 µM primers, 0.25 µM of each probe and 10 µl DNA samples. Amplifications were carried out using the following profile: 1 cycle at 95°C for 10 min, followed by 40 cycles of 95°C for 30 s, 60°C for 1 min. For syber-green amplification, a melting curve was added to show the amplification specificity. Amplification efficiencies were listed in Table 1. No qPCR inhibition was detected using 10^−3^ dilution of faecal samples using the Taqman exogenous internal positive control (Applied Biosystems), thus we used this dilution to perform all qPCR amplifications from faecal samples.

Bacterial genomic DNA of each reference strain (Table 1) was extracted as for faecal DNA and used to generate standard curves. Each reference strain was numbered from dilutions of each genomic DNA extract using an “All bacteria-qPCR” reaction with an *E. coli* standard curve (10-fold serial dilutions of genomic DNA from a culture numbered *E. coli* suspension). New DNA standard curves were obtained for each reference strain DNA (10-fold serial dilutions). Standard curve was generated by plotting cycle threshold (Ct) versus cell number of reference strain in the qPCR assay. Using cycle threshold (Ct) values in the linear range of the standard curve, faecal bacterial cells were interpolated from each standard curve generated in the same experiment. Then, results were expressed in bacterial cell/g faeces as mean and standard errors. When results were expressed in percentage of total bacteria, bacteria (expressed in bacterial cell/g faeces) was compared to all bacteria (expressed in bacterial cell/g faeces) and expressed as %.

### Statistical analysis

Data were expressed as mean ± standard deviation or percentage. Clinical and microbial data were compared between LA (Lactate Accumulator) and NLA (Non Lactate Accumulator) groups with Fisher exact test for qualitative variables and Mann Whitney test for quantitative variables. All statistical tests were two-sided. The critical level of statistical significance was set at p<0.05. Data were analyzed with the SAS 9.1 statistical software for windows (SAS Institute Inc., Cary. NC).

## Results

### SBS patient cohort

All 16 patients had undergone a major resection including a part of the jejunum, the entire ileum and part of the colon (Table 2). Aetiology of SBS was mainly venous/arterial mesenteric infarction (69%, n = 11/16). The study was performed 10±8 years following re-establishment of bowel continuity.

**Table 2:** Clinical characteristics of SBS patients.

Patient (sex)	Age when study (y)	Delay since continuity restablishment (y)	diagnosis	Remnant small bowel length (cm)	Remnant colon (%)
S1 (M)	63	3 months	Arterial mesenteric infarction	200	7
S2 (F)	75	10	Post-radiation enteritis	80	50
S3 (M)	41	1.5	Arterial mesenteric infarction	40	30
S4 (F)	63	1.3	Arterial mesenteric infarction	30	70
S5 (M)	52	21	Veinous mesenteric infarction	35	100
S6 (F)	62	12	Arterial mesenteric infarction	20	70
S7 (F)	75	15	Arterial mesenteric infarction	10	70
S8 (F)	43	12	Surgical complication	20	42
S9 (M)	63	1	Arterial mesenteric infarction	30	80
S10 (M)	34	1	Post-traumatic	50	40
S11 (F)	21	20	Caustic ingestion	30	50
S12 (M)	47	15	Veinous mesenteric infarction	40	50
S13 (M)	55	9	Arterial mesenteric infarction	15	42
S14 (M)	59	25	Arterial mesenteric infarction	50	70
S15 (M)	68	1	Arterial mesenteric infarction	25	50
S16 (F)	79	10	Post-radiation enteritis	70	70
Mean ±SD	**56±16**	**10±8**		**47±45**	**56±22**

Length of remaining colon in continuity was expressed in percent of usual length. Values are individual values.

Oral intake and macronutrient repartition are listed in Table 3. SBS patients had a total oral intake of 2661±1005 Kcal/day. The faecal wet weight was 2367±1140 g/day. The carbohydrate absorption (83±12%) was better than for proteins (42±13%) and fat (39±26%). BMI was 20.9±3.2 kg/m^2^ and levels of albumin, transthyretin protein and protein C reactive were normal (data not shown). Although S12 and S11 patients were known to be at risk for D-encephalopathy recurrent crisis, they did not present any clinical symptoms at the time of sampling.

**Table 3:** Intestinal absorption in SBS patients.

		Oral intake					Absorption (%)								
Patient (sex)	BMI Kg/m^2^	Kcal/day	Protein (g)	Fat (g)	Glucide (g)	Fibre (g)	Total	Protein	Fat	Glucide	HPN/wk	REE (Kcal/day)	Intake/REE	F.W (g/day)	HPN(%)
S1 (M)	24.1	2000	82	77	245	13	ND	ND	ND	ND	0	2436	0.8	3425	0
S2 (F)	16	1760	80	60	222	10	49	ND	ND	ND	4	1370	1.28	1800	64
S3 (M)	20.6	2971	108	142	246	14	63	46	52	87	4	2224	1.34	3425	67
S4 (F)	26.8	1914	72	68	209	12	53	42	31	73	6	1841	1.04	2985	67
S5 (M)	24.7	3440	118	125	382	22	ND	ND	ND	ND	2	2535	1.36	ND	13
S6 (F)	20.3	1890	93	97	96	15	74	36	11	74	3	1765	1.15	2030	24
S7 (F)	19.6	1680	61	46	208	10	73	48	55	91	7	1492	1.13	1154	80
S8 (F)	19.1	1830	45	100	200	9	38	22	84	55	4	1767	1.04	1280	43
S9 (M)	21.6	3475	132	147	323	21	72	70	50	95	3	2125	1.64	1415	40
S10 (M)	23.8	2713	84	101	308	17	65	48	54	78	0	2737	0.99	2510	0
S11 (F)	17.8	4860	173	217	441	19	30	32	0	90	0	1911	2.54	3423	0
S12 (M)	18.4	4260	174	131	488	13	58	50	9	92	2	2238	1.9	2210	17
S13 (M)	20	1920	80	80	220	11	48	ND	ND	ND	5	1790	1.07	4960	70
S14 (M)	20.3	3690	141	139	380	20	ND	ND	ND	ND	0	2175	1.7	ND	0
S15 (M)	25.5	2315	75	80	270	12	68	31	48	94	3	1812	1.28	1420	47
S16 (F)	16.4	1867	49	83	194	10	63	ND	ND	ND	3	1373	1.3	1111	61
**Mean ±SD**	**20.9±3.2**	**2661±1005**	**98±40**	**105±42**	**277±104**	**14±4**	**58±13**	**42±13**	**39±26**	**83±12**	**3±2**	**1974±402**	**1.4±0.4**	**2367±1140**	**37**

BMI: body mass index (Kg/m^2^). Total oral intake was expressed in Kcal/d. the repartition of proteins, fat, glucides and fibers was expressed as percent of total oral intake (g/day). HPN, number of home parenteral nutrition infusion par week and expressed in %. REE: resting energy expenditure. Intake/REE: ratio between oral intake (kcal/d) and REE (kcal/d). FW: fecal weight (g).

### Composition of the faecal microbiota in SBS patients

**Figure 1:**
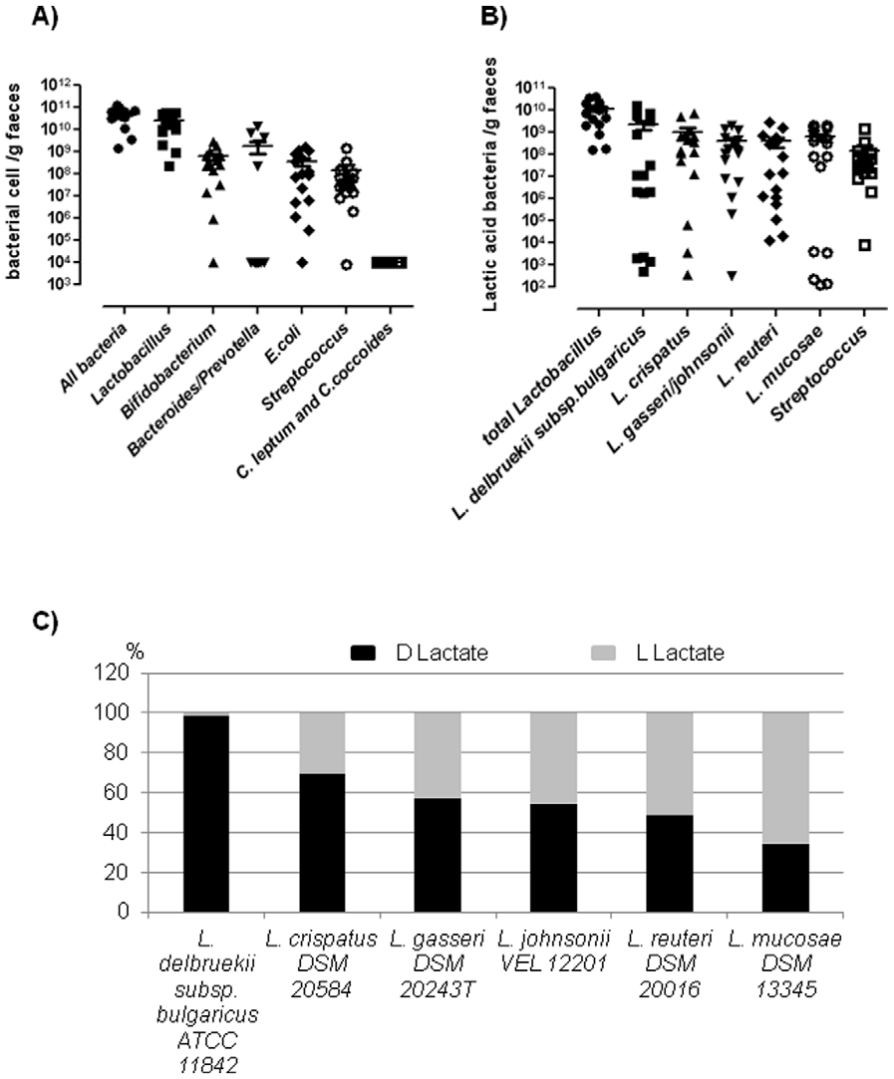
Composition of faecal microbiota in SBS patients and *in vitro* metabolic activity of lactic acid bacteria. A- Level of dominant and subdominant bacteria in the SBS faecal microbiota (expressed as the mean of bacterial cell/g faeces, mean value and individual values). *Lactobacillus* represented the *Lactobacillus/Leuconostoc* group determined by qPCR. B- Level of different lactic acid bacteria species in SBS faecal microbiota (lactic acid bacteria/g faeces, mean value and individual values). C- *In vitro* D and L lactate production of different reference species of *Lactobacillus*, during 24 hours (results were expressed in % of total D and L lactate in the medium).

In SBS faeces, total faecal bacteria represented 5.38×10^10^±3.4×10^10^ bacterial cell/g, where the *Lactobacillus/Leuconostoc group* was in majority (2.5×10^10^±2.1×10^10^ bacterial cell/g corresponding to 44±24% of total bacteria, mean ± standard error of individual percentage) (Figure 1A). *C. coccoides, C. leptum* (2×10^4^±0.5×10^4^ bacterial cell/g) were the less abundant, they representing <1% of total bacteria. *Bacteroides/Prevotella* (1.8×10^9^±3.8×10^9^ bacterial cell/g) represented a mean of 14±32% of total bacteria and was at variable levels among patients with low level (10^4^ bacterial cell/g) in 69% patients (11/16) and high level (2×10^8^ to 2×10^10^ bacterial cell/g) in 31% patients (5/16). Other lactic acid bacteria, *Bifidobacterium* and *Streptococcus* groups were in equivalent amount in the faeces 6.1×10^8^±7.7×10^8^ bacterial cell/g (5±6% of total bacteria) and 1.5×10^8^±3.7×10^8^ bacterial cell/g (2±3% of total bacteria) respectively. The *E coli* group represented 3.7×10^8^±5.1×10^8^ bacterial cell/g (3±4% of total bacteria) in the SBS faeces.

To determine the species in the SBS-related population; we sequenced the amplicon obtained by PCR from faecal DNA amplification by using universal *Lactobacillus/Leuconostoc* primers. This amplicon was mainly composed by *L. delbrueckii* subsp. *bulgaricus*, *L. crispatus*, *L. gasseri*, *L. johnsonii*, *L. reuteri*, *L. mucosae*. Therefore, we designed specific qPCR conditions and primers (Tables 1 and S1) for these *Lactobacillus* species. In all SBS patients, these species were indeed detected with a high inter-individual variability (Figure 1B). *L. mucosae* that we previously described in SBS patients [9], was present from 1.2×10^2^ to 2.0×10^9^ bacterial cell/g. Over a period of 8 months, faecal microbiota was stable for both dominant groups and the different species of *Lactobacillus* in S11 patient (Figure S1A and S1B). *L. mucosae* was detected at similar level in S12 patient over 4 years (data not shown). Thus, the overall composition of faecal microbiota seems to be specific for each patient and rather stable over time.

Each *Lactobacillus* species identified were characterized for its specific fermentative activity. It was monitored *in vitro* by the total amount of D and L-lactates expressed in percentage of total lactates (Figure 1C) or expressed in mM in the *in vitro* culture medium (Figure S2). *L. delbrueckii* subsp. *bulgaricus* synthesized *in vitro* specifically D-lactate (up to 70 mM). *L. mucosae* produced *in vitro* 30 mM D-lactate and 57 mM L-lactate, which respectively accounted for 35 and 65% of total lactate. Thus, each patient was characterized by the diversity of lactic acid bacteria in their faeces, and each lactic acid bacteria had a specific lactate production profile *in vitro* (Figure 1C).

### Faecal lactates amount in SBS patients

**Figure 2:**
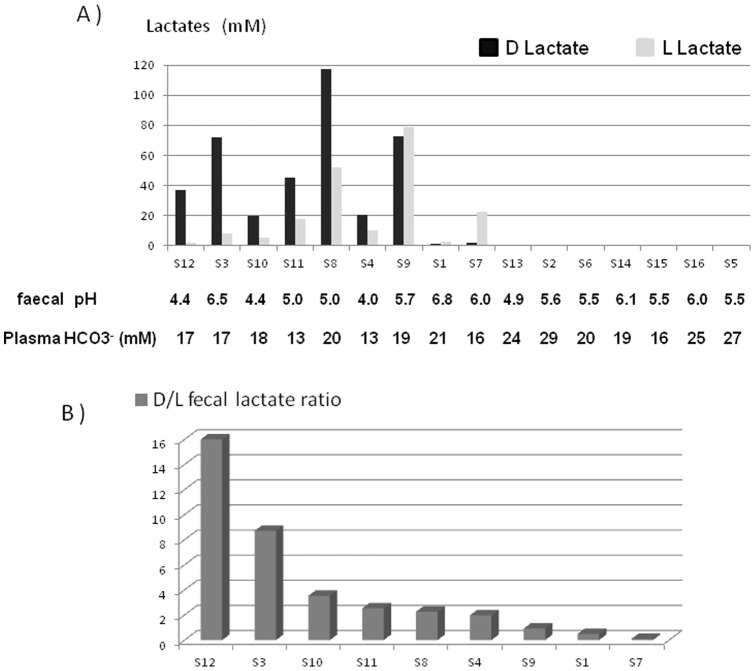
D and L-Lactates in faeces of SBS patients. A- Concentration of D and L lactate (mM) in SBS faeces revealed two groups : the faecal lactate-accumulator group (LA) and the non-accumulator group (NLA,). Individual faecal pH and plasma HCO3^–^ concentration (mM) was indicated. B- Faecal D/L lactate ratio in the SBS LA group of SBS patients.

The total amount of faecal lactates, the ratio of D/L lactates isoforms, faecal pH and the plasma concentration of HCO3^−^ are presented for each patient in Figure 2. Faecal pH values were in acidic range from 4.4 to 6.8. Of 16 patients, 9/16 (56%) accumulated lactate in their faecal samples (S12, S3, S10, S11, S8, S4, S9, S1, S7) (Figure 2A). In the faeces, the total amount of D-lactate ranged from 2 to 110 mM and the L-lactate ranged from 2 to 80 mM. The presence of lactates in faeces was used as criteria to define the Lactate-accumulator group (LA). In this LA group, 6/9 (67%) patients preferentially accumulated the D lactate isoform (with a D/L lactate ratio >1). In S12, we observed over 3 years a high amount of faecal D and L lactate with a D/L lactate ratio >2 (Table 4). S12 and S11, which are known to be at risk for D-encepahlopathy belong to the LA sub-group. S12 and S11 had a D/L lactate ratio above 2, and their plasma HCO3^−^ concentrations were respectively 17 and 13 mM (Figure 2). S13, S2, S6, S14, S15, S16, S5 had too lower level of lactates to be detected in their faecal sample, this sub-group (7/16, 44%) was defined as the non-lactate-accumulating group (NLA).

**Table 4:** Faecal D and L lactate concentrations (mM) in S12 patient over time from sequential sampling (T0, T0+3 years and T0+3.5 years).

Faeces	T0	T0+3 years	T0+3.5 years
**L Lactate mM**	**2.2±**0.8	**7.1±**0.9	**18.7±**1.2
**D Lactate mM**	**37.0±**1.3	**22.4±**0.6	**55.3±**3.5
**D/L Lactate ratio**	**16.5**	**3.2**	**2.9**

### Comparison between LA and NLA sub-groups

**Figure 3:**
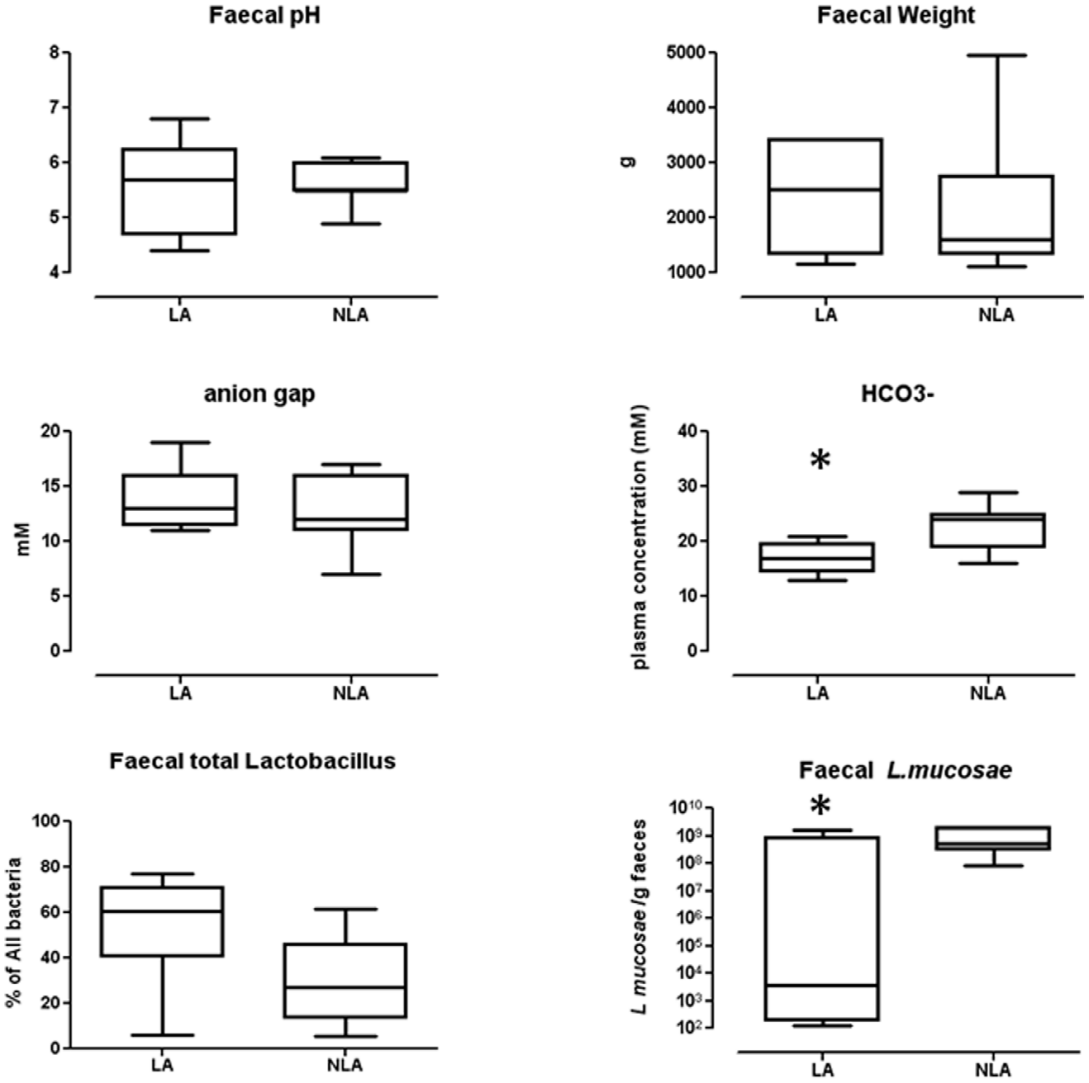
Different faecal and plasma parameters that characterize the lactate-accumulator group (LA) and the non-lactate accumulator group (NLA) of SBS patients. * p<0.05. Faecal total *Lactobacillus* was expressed in % of all bacteria. Faecal weight was expressed in g/24 h. Faecal *L. mucosae* was expressed as bacterial cell/g faeces. Plasma parameters: HCO3^−^ and anion gap were expressed in mM.

Between both LA and NLA groups, there were no significant differences of all clinical data listed in Tables 2 and 3, such as sex, age, remnant small bowel length, remnant colon and nutritional parameters such as oral intake, caloric and nutrients absorption and home parental nutrition supply. Although it tended to be higher in the LA group, the percentage of faecal total *Lactobacillus* was not statistically different in LA and NLA groups (54.9±23.1% *vs* 30.1±19.1%) (Figure 3). Faecal pH (5.5±0.8 *vs* 5.5±0.4), faecal weight (2425±955 g *vs* 2122±1426 g), and the anion gap (13.8±2.7 *vs* 12.6±3.4) were not statistically different in LA and NLA groups (Figure 3). On the other hand, the LA group had a lower concentration of plasma HCO3^−^ (17.1±2.8 mM) than the NLA group (22.8±4.6 mM) (p<0.01). In addition, the faecal D-lactate and L-lactate amounts were inversely correlated to plasma HCO3^−^ in the entire cohort of 16 patients (respectively spearman correlation −0.50, p = 0.04 and −0.53, p = 0.03). We did not find any correlation between faecal D or L-lactate amount and the different faecal bacteria except for *L. mucosae* that was significantly (p = 0.03) more present in the NLA (12.7±11.7% of *Lactobacillus/Leuconostoc*) *vs* LA group (2.8±4.7% of *Lactobacillus/Leuconostoc*) (Figure 3).

## Discussion

We recruited 16 type II SBS patients and described their nutritional and pathophysiologic status, their absorptive capacity, the composition of their faecal microbiota, the amount of faecal D/L lactate and routine serum values.

In healthy human stools, we have early described that microbiota is characterized by high dominant anaerobic bacteria (*C. leptum* 4.1×10^9^±3.9×10^9^bacterial cell/g faeces, *C. coccoides* 3.1×10^9^±2.9×10^9^ bacterial cell/g faeces and *Bacteroides/Prevotella* 7.9×10^8^±7.8×10^8^bacterial cell/g faeces) and sub-dominant bacteria (*Bifidobacteria* 2.8×10^8^±2.7×10^8^bacterial cell/g faeces, *Lactobacillus/Leuconostoc* 3.7×10^7^±3.4×10^7^bacterial cell/g faeces, and *Enterobacteria*) [9]. In contrast, SBS patients harboured a specific faecal microbiota that we called “lactobiota” because it is enriched in the *Lactobacillus/Leuconostoc group* and depleted in anaerobic micro-organisms (especially *Clostridium*) [7], [8], [9]. The number of patients enrolled in this study was comparable to our previous work [9], [22]. In both cohorts (see [9], [22] and Tables 2 and 3), SBS patients displayed similar clinical characteristics: they adopted a hyperphagic behavior (intake/REE>1) and they had similar absorptive capacities with better absorption of carbohydrates. Although stool samples were collected after a PEG treatment in the first cohort [9], [22] and without a PEG treatment in this study, we confirmed a shift in microbial communities between dominant and sub-dominant groups in SBS patients. Compared this cohort with that previously described [9], [22], both cohorts presented similar percentage of microbial groups and the emergence of *L. mucosae* in their lactobiota.

Lactobiota plays a crucial role in the digestion process mainly through *Lactobacillus*-related fermentative activities [40]. Although all patients are rich in *Lactobacillus/Leuconostoc group*, only 56% displayed an accumulation of faecal lactate. We thus defined two sub-groups of SBS patients: those accumulating lactate (LA) and those who did not accumulate lactate (NLA). In the cohort previously studied [9], [22], we also measured faecal lactate. Of 12 patients described in Joly *et al* [9], [22], 6 (50%) accumulated D and L-lactates in their faeces with a total amount of D-lactate ranging from 6 to 25 mM and the L-lactate ranging from 3 to 23 mM. LA and NLA groups of SBS patients are found in our both distinct cohorts. Since faecal *L mucosae* is less present in the LA subgroup, we could hypothesize that LA and NLA subgroups defined specific and different ecologic niches. Bustos *et al.* also described relatively high concentrations of faecal D-and L-lactate in some paediatric and adult SBS patients, who did not develop metabolic D-lactic acidosis [41]. Thus, LA and NLA groups of SBS that are also distinguished by their mean serum HCO3^−^, may constitute clinically relevant sub–types revealing two distinct metabolic signatures.

Lactate is produced both by eukaryotic and prokaryotic cells as a final metabolite of fermentation. In healthy humans, lactate produced as a key intermediate for the overall gut fermentation process isn't accumulated in faeces, it is absorbed by intestinal cells [42] or is used by lactate consuming bacteria [43], [44], [45], [46]. We found, coherently with the literature, that faecal D-lactate was undetectable in healthy human faeces and the faecal L-lactate was around 1.5 mM [41], [47], [48]. D-lactate enantiomer is toxic for neurons in a manner that is independent of acidosis [49] while L-lactate is metabolized at high level by neurons. D-and L-lactate share similar pathways of transport and metabolism in the digestive tract, but in human brain tissue, D isoform seems to impair the L lactate availability, thus provoking energy deficit in neurons [23], [50]. In SBS, the lactate accumulation in faeces could result, non-exclusively from 1) a predominance of lactate-producing bacteria over lactate-consuming ones, 2) an intense fermentation activity of lactobiota, 3) an imbalance between microbial production and intestinal absorption 4) and individual capacity of lactate metabolism. Lactate concentration in the colon depends mostly on microbial formation (main input) and microbial utilization or absorption from the host (main output). So, a lactate accumulation reflects an imbalance among these factors. As Duncan *et al* reported [51], D-L lactate induce >90% growth inhibition of dominant bacteria (such as *B. thetaiotaomicron*) at low pH, so the link between the accumulation of faecal lactate and the composition of lactobiota remains to be deeply explored by a deep sequencing. It could also be interesting to specifically quantify lactate consuming bacteria, such as *Eubacterium hallii*, *Anaerostipes caccae*, *Coprococcus catus*, bacteria related to *Megasphaera elsdenii*, *Eubacterium limosum* and *Anaerostipes coli* [52], [53], [54], [55], [56]. All these lactate consuming bacteria (mainly *Clostridium* cluster XIVa), representing around 10^7^ bacterial cell/g faeces (2 to 3% of the total bacteria) in healthy adult humans are highly oxygen-sensitive anaerobes [45], [52], [56]. In our study, *C. coccoides* group represented only 10^4^bacterial cell/g faeces in microbiota of all SBS patients (Figure 1), thus, these lactate consuming bacteria were under the detection threshold by PCR amplification (note that in our PCR conditions, the probes *C. coccoides* detect 3/6 of these lactate consuming bacteria [34]). In SBS patients, considering the short length of remnant small intestine and colon, it can be supposed that the level of oxygen might be too high to favor growth of strict anaerobic bacteria. The low faecal pH of SBS patients may also participate to the selection of growth and activities of some lactate consuming bacteria [57]. Even if lactate consuming bacteria were present in extremely low amount, would they be sufficiently active to decrease faecal lactates amount? In this context, it could be informative to evaluate, *in vitro*, the potential of lactate metabolism from fresh faecal microbial population in SBS patients.

In the LA sub-group, the total amount of lactate and the D/L ratio varied in each individual. S8 and S9 patients had the higher total amount of lactate, but the ratio between D/L was low (<1). On the contrary S12, S3, S10 and S11 preferentially accumulated D-lactate with a D/L lactate ratio over 2. This ratio remained over 2 during three years in S12 patient. Compared to healthy humans where plasma D-lactate concentration was undetectable (personal communication and [25]), plasma D and L-lactate concentrations in S12 patient were respectively 0.39±0.03 mM and 2.26±0.06 mM, and were stable overtime without D acidosis crisis (+3.5 years from this study). S12 and S11 had no D-acidosis crisis at the moment of the faecal sampling, but they were known to be at high risk for D-lactic acidosis. So, S12 and S11 patients, who preferentially accumulated D lactate in faeces, were at risk of D-encephalopathy crisis. We observed in our study an inverse correlation between plasma HCO3^−^ and faecal lactate levels. Furthermore, all patients who did not accumulate lactate and those who accumulate preferentially L isoform did not develop D-acidosis. In our previous study [9], [22], patients having a D/L ratio under 1 did not either develop D-encephalopathy (data not shown). D-lactate accumulation in faeces seems to indicate a microbiota dysbiosis that may be associated with a higher risk of metabolic acidosis and D-encephalopathy.

The D/L faecal lactate ratio seems to be the most relevant index first for an imbalanced microbiota in SBS patients and second for a higher D- encephalopathy risk, rather than D- and L-lactate faecal concentrations *per se*. Because D lactate amount in blood is not routinely dosed at hospital, we emphasize that the faecal amount of D and L lactate will be a useful tool to detect disturbed activity or composition of intestinal faecal microbiota after intestinal surgery in clinical management of short bowel syndrome or intestinal bypass [24]. To be more informative on the *in vivo* metabolic efflux, in the future, we will study the D-and L-lactate in faeces, blood and urine, in a longitudinal study, in a larger population of SBS patients who develop recurrent episodes of D-encephalopathy.

## Conclusions

We put in evidence a connection between intestinal dysbiosis and metabolite changes in the faeces of SBS patients. The plasma HCO3^−^ value, the faecal lactate amount and the faecal D/L lactate ratio constitute clinically relevant parameters that allow a classification of two sub–types of SBS patients: those having no detectable lactate in faeces (NLA sub-group), and those accumulating faecal D and L lactate in faeces (LA sub-group). In the LA sub-group, patients with high faecal D/L lactate ratio and low plasma HCO3^−^ value were at risk to develop recurrent D-encephalopathy episodes. All these criteria may be useful for identifying nutritional strategies to prevent or alleviate the deleterious consequences of D lactate accumulation in faeces in short bowel disease.

## Supporting Information

Figure S1 **Faecal microbiota of S11 patient over time from sequential collection (T0, T0+7 months and T0+8 months).** A- Dominant faecal microbiota in S11 patient was stable over 8 months; B- *Lactobacillus* species were relatively stable in S11 faeces over 8 months.
(TIF)

Figure S2 ***In vitro*** **D-and L-lactate production of different reference species of** ***Lactobacillus*** **, during 24 hours.** Results were expressed in mM.
(TIF)

Table S1 **Specificity of** ***Lactobacillus*** **specific primers used in this study.** Specificity of primers designed for this study was tested by performing: i) real-time qPCR on related and unrelated strains using a dilution 100 for DNA. Each primer set was named for its intended target bacteria. **+++**, Ct<20; **++**, Ct<25; **+**, Ct>25; **+/−**, Ct>30; **−**, no Ct detected; nd , not done; and ii) PCR amplification against all groups and species and detection of the amplicon on an 1.5% agarose gel.
(DOC)
