# Supplementary figures and images for: Faecal D/L Lactate Ratio Is a Metabolic Signature of Microbiota Imbalance in Patients with Short Bowel Syndrome
Source: PLoS One. 2013 Jan 23;8(1):e54335. doi: 10.1371/journal.pone.0054335 (PMC3553129; doi:10.1371/journal.pone.0054335)

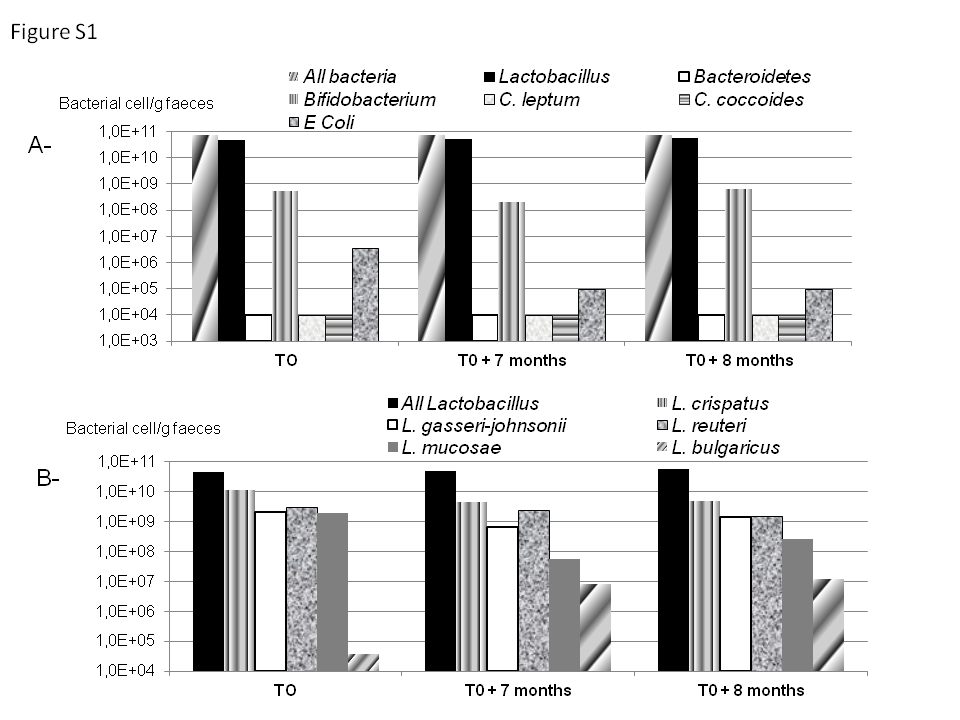

Supplement: Figure S1 — Faecal microbiota of S11 patient over time from sequential collection (T0, T0+7 months and T0+8 months). A- Dominant faecal microbiota in S11 patient was stable over 8 months; B- Lactobacillus species were relatively stable in S11 faeces over 8 months. (TIF) [file pone.0054335.s001.tif]
