# Supplementary material for: Faecal D/L Lactate Ratio Is a Metabolic Signature of Microbiota Imbalance in Patients with Short Bowel Syndrome
Source: PLoS One. 2013 Jan 23;8(1):e54335. doi: 10.1371/journal.pone.0054335 (PMC3553129; doi:10.1371/journal.pone.0054335)
Supplement: Table S1 — Specificity of Lactobacillus specific primers used in this study. Specificity of primers designed for this study was tested by performing: i) real-time qPCR on related and unrelated strains using a dilution 100 for DNA. Each primer set was named for its intended target bacteria. +++, Ct<20; ++, Ct<25; +, Ct>25; +/−, Ct>30; −, no Ct detected; nd , not done; and ii) PCR amplification against all groups and species and detection of the amplicon on an 1.5% agarose gel. (DOC) [file pone.0054335.s003.doc]

Table S1

| **Species** | **strain** | ***L. mucocase*** | | ***L. bulgaricus*** | ***L. crispatus*** | ***L. gasseri/***  ***johnsonii*** | ***L. reuteri*** |
| --- | --- | --- | --- | --- | --- | --- | --- |
| ***Clostridium leptum*** | DSM 753 | | - | - | - | **-** | - |
| ***Clostridium coccoides*** | ATCC 29236 | | - | - | - | **-** | - |
| ***Bacteroides fragilis*** | DSM 2151T | | - | - | - | - | - |
| ***Bifidobacterium adolescentis*** | DSM 20083 | | - | - | - | **-** | +/- |
| ***Escherichia coli*** | UEPSD S123 | | - | - | - | - | +/- |
| ***Lactobacillus acidophilus*** | VEL 12085 | | - | - | +/- | - | +/- |
| ***Lactobacillus acidophilus*** | ATCC 4356 | | +/- | +/- | +/- | - | +/- |
| ***Lactobacillus casei*** | BL 23 | | - | - | - | - | - |
| ***Lactobacillus crispatus*** | DSM 20584 | | - | - | **+++** | - | - |
| ***Lactobacillus delbrueckii* subsp. *bulgaricus*** | ATCC 11842 | | - | **+++** | - | **-** | +/- |
| ***Lactobacillus delbrueckii* subsp. *bulgaricus*** | VEL 12236 | | - | - | - | +/- | - |
| ***Lactobacillus fermentum*** | ATCC 14931 | | +/- | - | - | - | **++** |
| ***Lactobacillus gasseri*** | DSM 20243T | | **-** | - | - | **+++** | +/- |
| ***Lactobacillus jensenii*** | DSM 20557 | | - | - | - | **-** | - |
| ***Lactobacillus johnsonii*** | VEL 12201 | | **-** | - | - | **+++** | +/- |
| ***Lactobacillus mucosae*** | DSM 13345 | | **+++** | - | - | - | **++** |
| ***Lactobacillus paracasei*** | VEL 12237 | | - | - | - | - | - |
| ***Lactobacillus paracasei*** | VEL 12240 | | - | - | - | - | +/- |
| ***Lactobacillus plantarum*** | ATCC 14431 | | - | - | - | - | - |
| ***Lactobacillus reuteri*** | DSM 20016 | | +/- | +/- | - | - | **+++** |
| ***Lactobacillus salivarius*** | DSM 20555 | | - | - | - | - | - |
| ***Lactobacillus sakei*** | 23K | | - | - | - | - | - |
| ***Streptococcusus salivarius*** | ATCC 9222 | | - | - | - | +/- | - |
| ***Streptococcusus salivarius*** | DSM 20560 | | - | - | - | - | - |
